# Shock index creatinine: a new predictor of mortality in acute coronary syndrome patients

**DOI:** 10.1186/s12872-024-03730-4

**Published:** 2024-02-03

**Authors:** Widuri Wita Andriati Shariefuddin, Miftah Pramudyo, Januar Wibawa Martha

**Affiliations:** Department of Cardiology and Vascular Medicine, Hasan Sadikin General Hospital, Universitas Padjadjaran, Bandung, Indonesia

**Keywords:** Shock index creatinine, Global registry of acute coronary events score, Acute coronary syndrome, In-hospital mortality

## Abstract

**Background:**

The Shock Index Creatinine (SIC) scoring is a recently developed tool for risk stratification patients. These updated scoring was already used in ST-Elevation Myocardial Infarction (STEMI) patients. However its utility in predicting outcomes for patients with Acute Coronary Syndrome (ACS) remains unclear. This study aims to evaluate and update the current SIC score to predict in-hospital mortality among patients with ACS.

**Patients and methods:**

A retrospective cohort, Single-centered study enrolled 1349 ACS patients aged ≥ 18 years old diagnosed with ACS was conducted between January 2018 to January 2022 who met for inclusion and exclusion criteria. Study subjects were analyzed for in-hospital mortality and evaluated using binary linear regression analysis. The area under the curve (AUC) of SIC score was obtain to predict the sensitivity and specificity.

**Results:**

Multivariate analysis showed that SIC score was significantly associated with in-hospital mortality. High SIC score (SIC ≥ 25) had significantly higher in-hospital mortality (*p* < 0.001) with odds ratio for (95% CIs) were 2.655 (1.6–4.31). Receiver operating characteristics (ROC) curve analysis determine the predictive power of SIC score for in-hospital mortality. SIC had an acceptable predictive value for in-hospital mortality (AUC = 0.789, 95% CI: 0.748–0.831, *p* < 0.001). The SIC score for sensitivity and specificity were, respectively, 71.5% and 74.4%, with optimal cutoff of SIC ≥ 25.

**Conclusion:**

SIC had acceptable predictive value for in-hospital mortality in patients with all ACS spectrums. SIC was a useful parameter for predicting in-hospital mortality, particularly with a score ≥ 25. This is the first study to evaluate SIC in all spectrums of ACS.

## Background

Acute Coronary Syndrome (ACS) is a group of clinical manifestations characterized by an acute reduction in blood flow to the heart leading to myocardial ischemia [1, 2]. ACS has high morbidity and mortality rates. In 2019, the World Health Organization (WHO) reported that ischemic heart disease was the leading cause of mortality and responsible for 8.9 million death worldwide, with more than a third occurring in low- to middle-income countries [3]. Coronary heart disease (CHD) causes over 1,8 million fatalities per year in Europe, or almost 20% of all deaths [4].

ACS has been a major issue in most Asian countries due to its high mortality rates. Central Asian countries have the highest mortality rates, followed by West Asia, South Asia, and Southeast Asia [5]. The in-hospital mortality rate for ST-elevation myocardial infarction (STEMI) is very high. The mortality rate for non-ST-elevation myocardial infarction (NSTEMI) or stable angina pectoris is comparable to that of STEMI at 1 month to 1 year [6].

Several factors contribute to the high mortality rate of ACS patients. These include age, Killip class, risk factors such as kidney disorders, diabetes mellitus and previous myocardial infarction as well as PCI availability. Some authors have attempted to evaluate the predictor of mortality in ACS. Currently, the Thrombolysis in Myocardial Infarction score (TIMI) and the Global Registry of Acute Coronary Events score (GRACE) are the most commonly used tools for risk stratification [7].

The Shock Index (SI) and Modified Shock Index (MSI) are new score predictors that have emerged as simpler and easier tools for predicting mortality in ACS [8]. SI and MSI are now used in the emergency settings, including ACS patients [9–11]. In a study comparing MSI with SI, it was found that MSI was more accurate than SI for predicting mortality and major cardiovascular events in patients with NSTEMI who received percutaneous coronary intervention (PCI) [12].

The shock index creatinine (SIC) score is calculated using the following formula: (SI×100)–estimated clearance ratio (CCr). Shock index score was calculated using the following formula: heart rate (bpm)/systolic blood pressure (mmHg). The SIC is proposed as a new predictor of mortality because prior tests such as GRACE, CRUSADE, and Mehran scores indicate that kidney function is an important factor that can influence mortality prediction [13, 14].

Accurate mortality predictor is expected to improve and optimize management strategies for patients with ACS. The high mortality rate among ACS patients in Indonesia necessitates a novel approach for assessing prognosis and risk stratification. The SIC score, which includes an evaluation of kidney function, has been applied to STEMI population to predict in-hospital mortality. However, studies on SIC score in the ACS population has never been conducted. Thus, the application of the SIC score with the additional evaluation of renal function is expected to better predict mortality in ACS patients.

## Materials and methods

### Study design and patient selection

This study is a retrospective, single-center cohort analysis of all patients aged 18 years and older diagnosed with ACS and hospitalized at Dr. Hasan Sadikin General Hospital in Indonesia between January 2018 and January 2022. The study was approved by the Medical Research Ethics Committee of Dr. Hasan Sadikin General Hospital and informed consent was obtained from all participants. Data collected included patient demographics, medical history, laboratory results, adverse events, and medical treatment. Vital signs such as blood pressure and heart rate were recorded upon admission and alsoSI: Heart rate (bpm)/systolic blood pressure (mmHg). Estimated creatinine clearance rate (CCr) was computed using the published Cockcroft-Gault equations: man: (140–age)/Scr; woman:(140–age)/Scr 0.85 [13]. (SIx100)–estimated CCr was used to calculate the SIC. SIC was measured upon admission but did not contribute to deciding the patient’s treatment.

Data were collected from patients with acute coronary syndrome who came to emergency department of the Dr. Hasan Sadikin General Hospital Bandung and were included into One-ACS Registry. Patients with final diagnosis of acute coronary syndrome (including unstable angina pectoris (UAP), non-ST elevation myocardial infarction (NSTEMI), or ST-elevation myocardial infarction (STEMI)) were included. The diagnosis of ACS was based on ECG and cardiac enzyme criteria defined by the European Society of Cardiology. Patients who had incomplete data were excluded from the study. PCI was prioritized to all patients, however, in some condition where door to wire time exceeded 90 min, fibrinolytic was preferred for such patients. All patients using second generation drug-eluting stent (DES; including the XIENCE Everolimus-Eluting Stent (Abbott), Resolute Onyx Zotarolimus-Eluting Coronary Stent (Medtronic), Resolute Integrity Zotarolimus-Eluting Coronary Stent (Medtronic), The Ultimaster Sirolimus-Eluting Stent (Terumo). Pharmacological treatment was given to all patient according to the ESC guideline if not contraindicated.

### Statistical analysis

All statistical analyses were done using version 23.0 of SPSS (IBM Corp., Armonk, NY) and. The Kolmogorov-Smirnov test was used to analyze data distribution. Numerical variables with parametric distributions were provided as the mean standard deviation (SD), while those with non-parametric distributions were presented as the median and interquartile range. For categorical variables, total numbers and proportions were supplied. The Mann-Whitney U test was used to compare two numerical variables. As noted, we utilized the Chi-Square test or Fisher’s exact test to assess the differences between two categorical variables. ROC analysis was also performed to assess the accuracy of SIC scores in predicting in-hospital mortality. Further analysis using linear regression were performed to determine correlation between SIC and in-hospital mortality.

### Primary outcomes

This study’s primary outcome was in-hospital mortality, which was defined as all ACS patients who died in the hospital before discharge, irrespective of the cause of death.

## Results

### Baseline characteristics

A total of 1,443 patients with acute coronary syndrome admitted to our institution were included in the study. 94 patients were excluded due to incomplete data. Thus, total 1,349 patients were included in this study. The median of age was 58 years and 1.032 (76.5%) of the population were male. Table 1 demonstrated the baseline characteristics of total patients and divided into two groups based on in-hospital mortality. Analysis of significance was conducted using Chi-square analysis and Mann-Whitney U test. During the median length of hospitalization stay of 5 [3–6] days, 144 (10.7%) patients died.

**Table 1:** Baseline characteristics

Variable	Total (*n* = 1349)	Outcome		***p***- Value
		In-hospital mortality (*n* = 144)	Survive (*n* = 1205)	
**Demographic and lifestyle**				
Age (years), median (IQR)	58 (15)	64 (17)	57 (14)	**< 0.001**
Male, n (%)	1032 (76.5)	95 (66.0%)	937 (77.8%)	**0.002**
**Smoking status**				
Current, n (%)	802 (59.5)	71 (49.3%)	731 (60.7%)	**0.009**
**Risk factors**				
Hypertension, n (%)	847 (62.8)	99 (68.8%)	748 (62.1%)	0.120
Type II DM, n (%)	293 (21.7)	40 (27.8%)	253 (21.0%)	0.062
Dyslipidemia, n (%)	263 (19.5)	29 (20.1%)	234 (19.4%)	0.360
Family history of premature CAD, n (%)	132 (9.8)	14 (9.7%)	118 (9.8%)	0.979
Obesity, n (%)^a^	89 (6.6)	5 (3.5%)	84 (7.0%)	0.110
Chest pain duration (hours)^b^	10 (19)	15 (32)	10 (16)	**< 0.001**
**Killip classification**				
Killip II, n (%)	218 (15.7)	25 (17.4%)	193 (16.0%)	**< 0.001**
Killip III, n (%)	35 (1)	12 (8.3%)	23 (1.9%)	**< 0.001**
Killip IV, n (%)	128 (9.4)	53 (36.8%)	75 (6.2%)	**< 0.001**
**ACS Types**				
STEMI, n (%)	781 (56.6)	90 (62.5%)	691 (57.3%)	0.594
NSTEMI, n (%)	487 (36)	48 (33.3%)	439 (36.4%)	0.594
UAP, n (%)	80 (5.1)	6 (4.2%)	74 (6.1%)	0.594
SBP (mmHg), median (IQR)	120 (30)	110 (50)	120 (30)	**< 0.001**
DBP (mmHg), median (IQR)	80 (20)	70 (20)	80 (20)	**< 0.001**
MAP (mmHg), median (IQR)^c^	91.3 (20)	83 (30)	93 (20)	**< 0.001**
Heart rate (bpm), median (IQR)	80 (26)	96 (30)	80 (24)	**< 0.001**
**Laboratory findings at admission**				
Direct blood glucose (mg/dL), median (IQR)	129 (58)	151 (95)	126(53)	**0.001**
Ureum (mmol/L), median (IQR)	32.9 (25)	48 (68)	31 (23)	**< 0.001**
Creatinine (µmol/L), median (IQR)	1.17 (17)	1.72 (1.47)	1.14 (0.57)	**< 0.001**
Creatinine clearance (mL/min), median (IQR)	59 (37)	32.9 (32.3)	60 (34.9)	**0.009**
Troponin-I (ng/L), median (IQR)	4.9 (10)	10 (9)	4.6 (10)	**0.028**
**Revascularization procedure**				
Fibrinolytic, n (%)	155 (10.6)	8 (5.6%)	147 (12.2%)	**0.047**
PCI, n (%)	868 (64.2)	62 (43.1%)	805 (66.8%)	**< 0.001**

**Abbreviations**: Abbreviations: ACS: acute coronary syndrome; CAD: Coronary artery disease; DBP: diastolic blood pressure; SBP: systolic blood pressure; IQR: interquartile range, PCI: percutaneous coronary intervention; MAP: mean arterial pressure; STEMI: ST-elevation myocardial infarction; NSTEMI: non-ST elevation myocardial infarction; UAP: unstable angina

^a ^Obesity was defined as body mass index of greater than 30 kg/m^2^

^b^ Chest pain duration was calculated since the onset of pain when the patient felt it before admission

^c^ MAP was calculated as: (SBP + 2 DBP)/ 3

### Determinant of in-hospital mortality

A correlation analysis was conducted to evaluate the determinant of mortality in ACS patients. Spearman correlation showed that almost all variable, except for sex, BMI, dyslipidemia, hypertension, diabetes mellitus, and ACS type were significantly associated with in-hospital mortality. However, only the SIC score, Killip class, and GRACE score had at least a weak correlation with in-hospital mortality (*r* > 0,3), while others had a very weak correlation (*r* < 0,3) (Table 2).

**Table 2:** Spearman correlation analysis on in-hospital mortality

Variables	***R*** value	***P*** value
Age	0.166	< 0.001
Sex	0.001	0.979
Smoking	0.071	0.009
SIC Score	0.309	< 0.001
Body Mass Index	-0.056	0.061
Systolic blood pressure	-0.155	< 0.001
MAP	-0.149	< 0.001
Heart Rate	0.186	< 0.001
MSI	0.221	< 0.001
SI	0.221	< 0.001
Urea	0.227	< 0.001
Creatinine	0.236	< 0.001
Troponin	0.081	0.005
GRACE	0.336	< 0.001
Hypertension	0.042	0.120
Dyslipidemia	0.007	0.810
Diabetes mellitus	0.051	0.062
Killip class	0.3	< 0.001
ACS type	0.035	0.204
Creatinine Clearance	-0.266	< 0.001

**Abbreviations:** ACS: acute coronary syndrome; GRACE: Global Registry of Acute Coronary Events; MAP: mean arterial pressure; MSI: modified shock index; SI: shock index; SIC: shock index creatinine

Univariate analysis showed SIC had a significant correlation with in-hospital mortality. Increasing SIC was associated with an increased likelihood of in-hospital mortality (Table 3). Furthermore, multivariate analysis was completed by adjusting several confounding factors with *p* < 0.25 and showed SIC was significantly associated with in-hospital mortality in patients with ACS. [OR = 3.223 (2.600-3.643), *p* value < 0.001]. Factors included in multivariate analysis were those with *p* value of < 0.25 in the univariate analysis, they are: age, sex, smoking status, hypertension, DM type 2, obesity, chest pain duration, Killip class, systolic BP, diastolic BP, mean BP, heart rate, blood glucose, ureum, creatinine, creatinine clearance, troponin, fibrinolytic, and PCI. Another multivariate analysis was conducted after excluding patients with cardiogenic shock, mechanical complication, and total AV block (*n* = 1,221). Adjusted OR showed significant association between SIC and in-hospital mortality [adjusted OR = 2.655 (1.633–4.315), *p* value < 0.001].

**Table 3:** Multivariate analysis for SIC score in all population (*n* = 1,349) and after adjustment by excluding patients with cardiogenic shock, mechanical complication, and total AV block (*n* = 1,221)

Variables	OR (95% CI )	***P*** value	Adjusted OR (95% CI)	***P*** value
SIC Score	3.223 (2.600-3.643)	< 0.001	2.655 (1.633–4.315)	< 0.001

**Abbreviations:** CI: confidance interval; OR: odds ratio; SIC: shock index creatinine

### ROC analysis of SIC, cutoff score determination for predicting in-hospital mortality

**Fig. 1:**
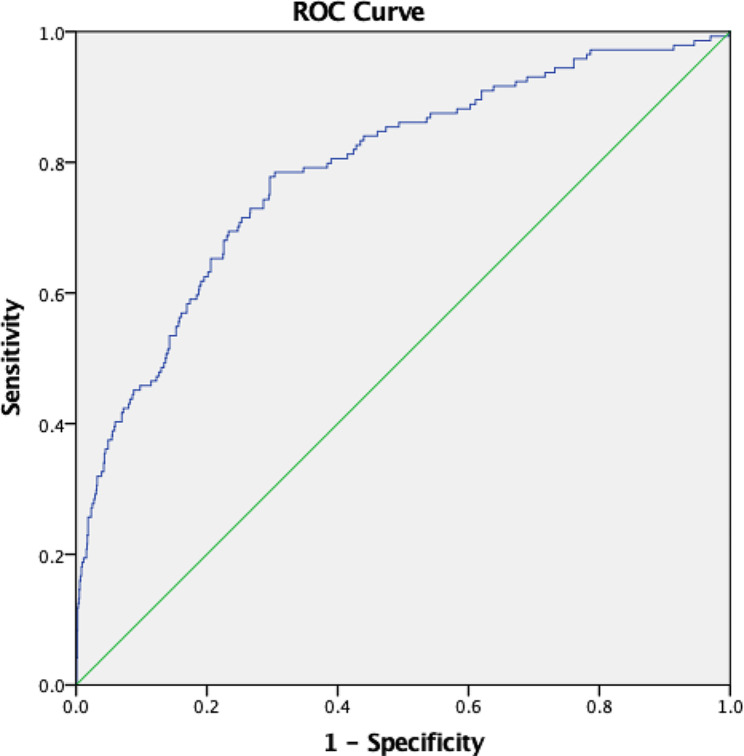
ROC curve in all population **Abbreviations:** ROC: Receiver operating characteristics; Green line: reference line; Blue line: Shock Index Creatinine

**Fig. 2:**
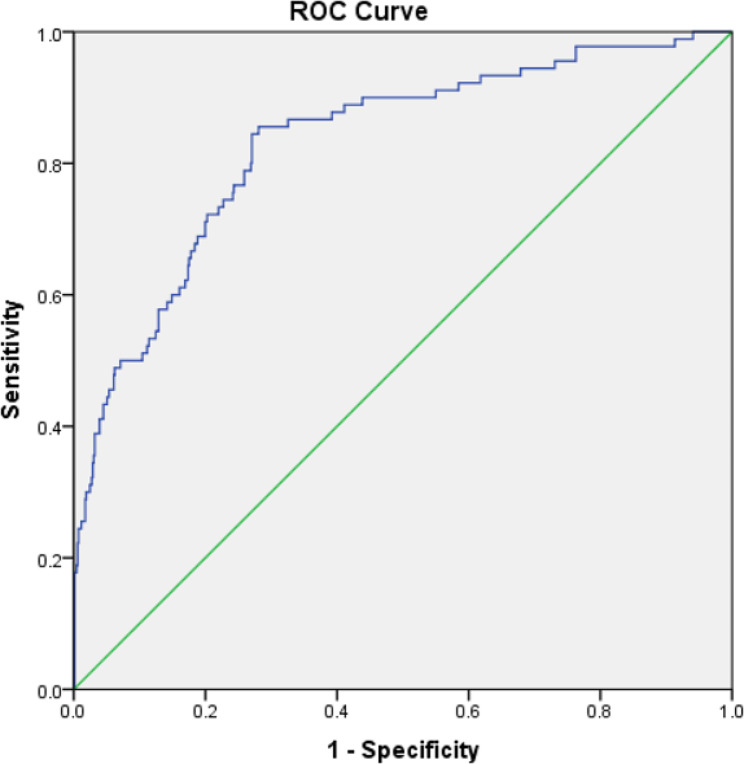
ROC curve in STEMI population **Abbreviations:** ROC: Receiver operating characteristics; Green line: reference line; Blue line: Shock Index Creatinine

The optimal SIC cutoff was determined using ROC curve analysis. This analysis revealed that the optimal cut-off for SIC was one based on a value that had the closest distance to the upper left corner of the ROC curve. SIC had an acceptable predictive value for in-hospital mortality (AUC = 0.789, 95% CI: 0.748–0.831, *p* < 0.001) (Table 4). SIC cutoff of 25 had a sensitivity of 71.5% and specificity of 74.4% for predicting in-hospital mortality (Fig. 1). The patients were divided into two groups based on the SIC category score cutoff of < 25 (*n* = 936) and the high SIC score ≥ 25 (*n* = 413) with significant in-hospital mortality low SIC and high SIC, respectively (*n* = 41 ; 103, *p* value < 0.001) (Table 5). The distribution of SIC among STEMI, NSTEMI and UAP group were shown in Table 6. Statistical analysis showed no significant difference of SIC among the 3 groups (*p* = 0.237). Furthermore, we conducted ROC analysis for STEMI group only and we found that SIC had better predictive value for in-hospital mortality (AUC = 0.831, 95% CI: 0.784–0.878, *p* < 0.001) (Table 7). In this population, SIC cutoff of 25 had a sensitivity of 74.4% and specificity of 76.8% for predicting in-hospital mortality (Fig. 2).

**Table 4:** ROC analysis in all population

Population	Area Under Curve	Std Error	95% Confidence Interval (CI)	
			Lower	Upper
SIC score	0.789	0.02	0.748	0.831

**Abbreviations:** ROC: Receiver operating characteristics; CI: confidance interval; SIC: shock index creatinine

**Table 5:** In-hospital mortality based on SIC cutoff score

Variables	In-hospital Mortality *N* = 144	***P*** value
SIC < 25	41 (28.5%)	< 0.001
SIC ≥ 25	103 (71.5%)	

**Abbreviations:** SIC: shock index creatinine

**Table 6:** SIC distribution among STEMI, NSTEMI, and UAP group

	Low SIC	High SIC
STEMI	554 (73.94%)	227 (29.06%)
NSTEMI	324 (66.6%)	163 (33,4%)
UAP	57 (71.3%)	23 (28,7%)

**Abbreviations:** STEMI: ST-elevation myocardial infarction; NSTEMI: Non-ST elevation myocardial infarction; UA: unstable angina; SIC: shock index creatinine

**Table 7:** ROC analysis in STEMI population

Population	Area Under Curve	Std Error	95% Confidence Interval (CI)	
			Lower	Upper
SIC score	0.831	0.024	0.784	0.878

**Abbreviations:** ROC: Receiver operating characteristics; CI: confidance interval; SIC: shock index creatinine

## Discussion

The SIC score is a combination of SI and renal function for the determining the prognosis of in-hospital mortality patients. This is the first study to utilize the SIC score for determination of in-hospital mortality in patients presenting with all spectrums of acute coronary syndrome. This study found that SIC had an acceptable predictive value for in-hospital mortality both as numeric or categorical variable (AUC = 0.789, 95% CI: 0.748–0.831, *p* < 0.001; AUC = 0.729, 95% CI: 0.684–0.774, *p* < 0.001 respectively). Chiang et al. also demonstrated that SIC had an acceptable predictive value for in-hospital mortality in STEMI patients (AUC = 0.792, 95% CI: 0.748–0.836, *p* < 0.001) [15]. Ran et al., found that the predictive value and calibration of SIC for in-hospital mortality was excellent in derivation [area under the curve (AUC) = 0.877, *p* < 0.001; Hosmer-Lemeshow chi-square = 3.95, *p* = 0.861] [13].

In this study, an SIC cutoff of 25 had sensitivity of 71.5% and specificity of 74.4% for predicting in-hospital mortality in ACS patients. This is the first study to evaluate SIC in all spectrums of ACS. A better sensitivity and specificity was found when the ROC analysis was conducted in STEMI population only (sensitivity of 74.4% and specificity of 76.8%). The high sensitivity and specificity was also found in the previous studies. Chiang et al., found that an SIC cut-off of 21.0 had sensitivity of 67.2% and specificity of 83.5% for in-hospital mortality of STEMI (in this study, SIC cut-off of 21 in STEMI population had sensitivity of 84.4% and specificity of 72.8% for in-hospital mortality) [15]. Ran et al., found a sensitivity of 82.4% and specificity of 77.8% for SIC cutoff of 10 in predicting in-hospital mortality (in this study, SIC cut-off of 10 in STEMI population had sensitivity of 86.7% and specificity of 62.4% for in-hospital mortality) [13]. Those findings indicate that SIC had good sensitivity and specificity for predicting in-hospital mortality in STEMI and all spectrums of ACS. Our results suggest that SIC is a rapid tool with good sensitivity and specificity for predicting in-hospital mortality in patients presenting with ACS.

ROC analysis in our study demonstrated that SIC had a good predictive value for in-hospital mortality. SIC had a better correlation to in-hospital mortality than SI and MSI score on spearman analysis. Our study also demonstrated that SIC had a significant association with in-hospital mortality both in univariate and multivariate analysis. Ran et al., also found that the discriminatory capacity of SIC for in-hospital mortality was non-inferior to the GRACE scale, but SIC was a better predictor than the TIMI risk score [13]. Chiang et al., also found that SIC had better predictive power than that of SI and MSI [15].

Decrement of parameters related to cardiac function such as cardiac index, stroke volume, and left ventricular (LV) stroke work developed during acute myocardial infarction, especially if cardiogenic shock. In other words, the heart’s ability to meet systemic perfusion needs is decreased substantially. Baroreceptors in the blood vessel wall stimulate the vasomotor areas in the brainstem to increase heart rate and arterial vasoconstriction in hypotensive conditions. A series of neurohumoral reactions are triggered following myocardial infarction, including activation of the sympathetic nervous system. The release of catecholamines will trigger an increase in blood pressure (BP) and heart rate (HR) to compensate for decreased cardiac output due to myocardial infarction. This initial compensatory system can be represented by the components of the shock index: pulse rate and blood pressure.

The index of BP and HR after myocardial infarction may reflect the cardiovascular system and neuroendocrine system’s condition as well as the patient’s hemodynamic status. It is a sensitive indicator of left ventricular dysfunction and the degree of hemodynamic stability rather than just relying on HR or SBP alone [16]. Several previous studies have shown that the shock index can be a predictor of major adverse cardiac events (MACE) or death in patients with ACS [17, 18]. Wang et al. have attempted to determine whether the prognostic value of the shock index and its derivatives (MSI, age SI [age x SI], age MSI [age x MSI]) is better than the TIMI risk index for predicting adverse outcomes in STEMI patients undergoing primary PCI. Multivariate analysis shows that high SI (and its derivatives) values are associated with higher complication rates [19].

Renal dysfunction is believed to be a risk factor for mortality in patients with myocardial infarction [4]. Cywinski et al. showed that estimated renal function was a better prognostic indicator than Scr [20]. CCr by Cockcroft- Gault has adequate discriminatory ability, with an AUC > 0.8 for prediction of poor outcomes, which was better than other equations for glomerular filtration rate estimation in patients with acute coronary syndrome [21]. Therefore, it can be concluded that the addition of CCr to SI could result in better predictive accuracy in patients with STEMI. SIC included 3 factors that were easily collected and calculated, in contrast to the GRACE score which included more variables [13]. The previous studies only evaluated the predictive value of SIC in the STEMI population, while in our study, patients with all ACS spectrums were included; adding unstable angina and NSTEMI patients into account [13, 15]. Our results showed that SIC is not only useful for predicting mortality in STEMI but also in all ACS spectrums.

The finding in our study suggested that SIC could be applied to stratify the risk of ACS patients and determine whether an interventional procedure is necessary. It may also help plan the appropriate time for coronary intervention upon the patient’s admission.

### Limitation

The limitation of this study was the retrospective design from a single center that requires further validation in a large-scale multicenter study. Further study is still needed to confirm the ability of SIC in predicting in-hospital mortality in ACS patients. Furthermore, our study did not evaluate the pharmacological treatment received by the subjects, and did not analyze the cause of death of the subjects.

## Conclusion

Our study demonstrated that SIC had acceptable predictive value for in-hospital mortality in patients with all ACS spectrums. SIC was a useful parameter for predicting in-hospital mortality, particularly with a score ≥ 25. The predictive value of SIC is acceptable for in-hospital mortality.

## Data Availability

Data sharing is available and can be contacted through corresponding email widuriwita@gmail.com.

## Abbreviations

SIC: Shock Index Creatinine
STEMI: ST Elevation Myocardial Infarction
ACS: Acute Coronary Syndrome
AUC: Area Under Curve
ROC: Receiving Operating Characteristics
WHO: World Health Organization
CHD: Coronary Heart Disease
NSTEMI: Non ST Elevation Myocardial Infarction
UAP: Unstable Angina Pectoris
TIMI: Thrombolysis in Myocardial Infarction
GRACE: Global Registry of Acute Coronary Events
SI: Shock Index
MSI: Modified Shock Index
PCI: Percutaneous Coronary Intervention
CCR: Creatinine Clearence Ratio
CRUSADE: Can Rapid Risk Stratification of Unstable Angina Patients Suppress Adverse Outcomes With Early Implementation
SD: Standard Deviation
CI: Confidence Interval
OR: Odds Ratio
LV: Left Ventricle
BP: Blood Pressure
HR: Heart Rate
SBP: Systolic Blood Pressure
DBP: Diastolic Blood Pressure
MAP: Mean Arterial Pressure
MACE: Major Adverse Cardiovascular Event
